# One Month of Classic Therapeutic Ketogenic Diet Decreases Short Chain Fatty Acids Production in Epileptic Patients

**DOI:** 10.3389/fnut.2021.613100

**Published:** 2021-03-29

**Authors:** Cinzia Ferraris, Erika Meroni, Maria Cristina Casiraghi, Anna Tagliabue, Valentina De Giorgis, Daniela Erba

**Affiliations:** ^1^Human Nutrition and Eating Disorder Research Center, Department of Public Health, Experimental and Forensic Medicine, University of Pavia, Pavia, Italy; ^2^Department of Food, Environmental and Nutritional Sciences DeFENS, University of Milan, Milan, Italy; ^3^Department of Child Neurology and Psychiatry, Istituto di Ricovero e Cura a Carattere Scientifico (IRCCS) Mondino Foundation, Pavia, Italy

**Keywords:** ketogenic diet, epilepsy, short chain fatty acids, fecal water toxicity, gut environment

## Abstract

Ketogenic diet (KD), a high fat and very low carbohydrates diet, is used worldwide for the treatment of drug resistant epilepsy but, due to its composition, it might exert an impact on gut health. Even though data of KD effects on intestinal microbiota changes are recently emerging, its influence on the gut environment has been scarcely addressed so far. The aim of this study was to investigate whether 1 month of KD affects the gut environment in epileptic patients, by analyzing short chain fatty acids (SCFA) production and fecal water toxicity. A total of seven patients were enrolled. Stool samples were collected before (T0) and after 1 month of KD (4:1 ketogenic ratio) (T1). SCFA were determined by GC-FID and fecal water toxicity in Caco-2 cell culture by comet assay. Concentrations of SCFA significantly decreased after KD (*p* < 0.05): in particular, we found a 55% reduction of total SCFA level, a 64% reduction of acetate, 33% of propionate, and 20% of butyrate (*p* < 0.05). Cytotoxicity of fecal water extracted from stool samples was not significantly altered by diet, while genotoxicity was slightly decreased after KD (*p* < 0.05). Genotoxicity values were consistent with data previously obtained from a healthy Italian population. The present study suggests that 1 month of KD significantly reduce SCFA production. Since SCFA produced by gut microbiota exert many health promoting effects on either the gut environment or human metabolism, these results open a new branch of investigation into KD effects.

## Introduction

The classic ketogenic diet (KD) is a normocaloric high-fat very low-carbohydrate diet, used worldwide for the treatment of drug-resistant epilepsy (DRE) for its anticonvulsant effect (1). KD is typically composed of a 4:1 ratio of fat (in grams) to protein plus carbohydrates (in grams), thus shifting the predominant caloric source from carbohydrates to fat (2).

This diet induces changes in gut microbiota that have been addressed *in vivo* in animal (3–5) and human (6–10) studies. Increase of specific genera, such as *Desulfovibrio* and *Akkermansia* (8, 11), decrease in the relative abundance of phylum, such as *Actinobacteria* and *Firmicutes*, and increase of *Bacteroidetes* and *Proteobacteria*, were displayed in patients after ketogenic dietary therapies (6).

Changes in the microbiota community potentially impact the microbial metabolome which, in turn, exerts influence on host physiology. For instance, short chain fatty acids (SCFA) production (namely acetate, propionate, and butyrate), resulting from microbial fermentation of dietary non-digestible carbohydrates, is affected by numerous factors including the source and the amount of available substrates, and the complex interaction between the gut, transit time, and ecological factors (12). SCFA, and butyrate in particular, are the main source of energy for colonocytes, and appear important in regulating the integrity of the epithelial barrier (13). The production of SCFA reduces intestinal pH, therefore preventing the growth of pH-sensitive pathogenic bacteria. Butyrate is also able to inhibit growth, induce terminal differentiation, and promote apoptosis in transformed cells possibly by the inhibition of histone deacetylase activity (14). Moreover, butyrate inhibits the activation of the transcriptional factor NF-kB and consequently the production of pro-inflammatory cytokines (15). At an organism level, several studies have shown that propionate and acetate can modulate the glycol-lipid metabolism, by inhibiting the hepatic synthesis of cholesterol and improving insulin sensitivity (16). With these multiple actions in mind, it is possible that altered SCFA production can negatively affect host's health either in the gut or at a systemic level.

By altering the gut microbial ecosystem, KD could also affect the composition of human feces, exposing colon mucosa to risk factors. A non-invasive way to study this potential risk is by investigating, *in vitro*, the genotoxicity of the aqueous extract from stool (fecal water), via single-cell gel electrophoresis (comet assay). This sensitive technique has been successfully applied to investigate the genotoxicity of specific compounds (17) or the effect on the gut environment due to different dietary interventions in humans (18). The few studies that have monitored the genotoxicity of fecal water in healthy volunteers (17, 19, 20) suggest that it could be considered as a predictive biomarker for intestinal carcinogenic risk.

On these grounds, our study investigates the hypothesis that 1 month of therapeutic KD by impacting the microbiota ecosystem, could affect microbial metabolites production and, consequently, the gut environment. To this aim, we analyzed the production of SCFA, and the toxicity of fecal water, in order to reach an integrated view of the KD impact on gut health.

## Methods

### Subjects

The KD effects were assessed through the enrolment of seven patients (four females and three males, ages ranging between 2 and 46 years) affected by DRE. From each patient, a fecal sample was collected before (T0) and after 1 month (T1) of treatment with a classic KD. Exclusion criteria included fecal incontinence, gastrointestinal disorders or the use of antibiotic or probiotic/prebiotic supplements during the month prior to the study, age <2 years old, and participants who were under- and overweight. The recruitment began in September 2017 and lasted until June 2018. In this period, 28 patients were diagnosed with DRE and prescribed a ketogenic diet; only seven were suitable for the study (16 had no fecal continence and 5 declined to participate). The study protocol complied with the principles of the Declaration of Helsinki, and was approved by the Ethical Committee of San Paolo Hospital—University of Milan (Comitato Etico Milano Area 1, Protocol number 2017/EM/147). Written informed consent to participate in this study was provided by the participants or participants' legal guardian/next of kin.

### Ketogenic Diet Treatment

Using a standardized approach (21), the Human Nutrition Research Center keto-team implemented a non-fasting dietary protocol—which was uniformly applied in all of the study patients—characterized by gradual increases of the ketogenic ratio. The usual caloric intake as well as food intolerances and preferences were investigated using weekly food diaries. Energy prescriptions were tailored to each patient's specific requirements (based on basal energy expenditures measured by indirect calorimetry and subsequently corrected for physical activity levels). KD plans with increasing ketogenic ratios were adjusted at the individual level by an experienced dietician. Macronutrient composition included a minimum of 0.8–1 g of proteins from animal sources (e.g., eggs, milk, meat, poultry, and fish) were supplied per kilogram of body weight. All of the study participants were prescribed sugar-free multivitamin and mineral supplements according to their age and sex. No probiotic or prebiotic supplementation was provided during the treatment. All patients were started at home on a 1:1 ketogenic diet, with ketogenic ratios subsequently increased to 2:1, 3:1, or 4:1 in order to achieve blood β-OHB levels ≥2.0 mmol/L. Family members were instructed to monitor blood β-OHB concentrations twice per day during the induction phase and report values by email to the study investigators.

### Collection and Preparation of Samples

Daily total stool was collected in disposable bedpans, immediately frozen, delivered to the laboratory within 24–48 h and stored at −80°C. Aliquots of stool were then divided for biological assessments and fecal water (FW) preparation. The humidity of the fecal samples was determined, after drying at 105°C overnight, by the weight difference of a sample aliquot according to the official AOAC method (22).

### Preparation of Fecal Water

From the frozen stool samples, FW was obtained after 2 h thaw at room temperature; samples were then diluted 1:1 (w/v) in sterilized PBS and homogenized manually until a uniform consistency was achieved. Samples were centrifuged at 24,000 rpm (35,000 × g) for 2 h at 20°C (Beckman L7-55 Ultracentrifuge; Beckman Limited, High Wycombe, UK) and the supernatants were carefully decanted and stored at −80°C (19). For the cytotoxicity and genotoxicity evaluations, the FW samples were rapidly defrosted, centrifuged at 11,000 rpm for 2 min at 20°C to remove any residuals, and filtered through a 0.45m filter (VWR International, USA).

### SCFA Measurement

SCFA concentrations were assessed in accordance with the method proposed by Weaver et al. (23), modified as follows. Stool (200 mg) were suspended in 1 ml of double distilled water, homogenized and, after 30 min, centrifuged (15,000 rpm) for 15 min at 10°C. Aliquots (500 μl) of supernatant were added with 200 μl of 85% orthophosphoric acid, 200 μl of 2% (v/v) sulphuric acid, and 100 μl of 10 mmol/L ethyl-butyric acid (Sigma-Aldrich, Italy) in 12% acetic acid as internal standard. SCFA were gently extracted for 1 min with 1 ml of ethyl-ether/heptane (1:1 v/v) and centrifuged for 10 min at 3,000 rpm. The organic layer was removed for analysis by a Varian 3400 CX gas liquid chromatograph equipped with a Varian 8200 CX auto sampler and a HP-FFAP fused-silica capillary column (30 m, 0.53 mm i.d. with a 1-mm film) as previously reported (24). Quantification of the SCFA was obtained through external calibration curves (concentration range 0.25–10 mmol/L). Results were expressed as mg/g of the dry weight of feces.

### Fecal Water Toxicity

#### Cell Culture

The intestinal epithelial cell line Caco-2, derived from a human colonic adenocarcinoma (HTB-37; ATCC, Manassas, VA), is a well-established and validated model of the human intestinal mucosa (25). Cells were cultured as previously described (19). The culture medium was routinely changed every 2 days and the day before exposure to FW. All cell culture reagents were purchased from Sigma–Aldrich (St. Louis, MO, United States) and chemicals were purchased from Merck (Darmstadt, Germany). For the analysis, cells were seeded on 60 mm plates (Cellstar, Greiner, Germany) at a density of 105 cells/cm^2^, and maintained for 10 d in complete medium; the medium was changed three times a week (25).

#### Cytotoxicity and Genotoxicity

A suspension of differentiated Caco-2 cells (3.5 × 10^5^ cells/mL in 380 μL) was incubated with FW (120 μL) or medium (120 μL negative control) or H_2_O_2_ 500 mmol/L (120 μL positive control) for 30 min at 37°C on a shaking platform (19). Every FW recovered from the stool samples was analyzed four times and controls (both negative and positive) were included in each batch. After incubation, an aliquot of cell suspension was used to assess the cytotoxicity of FW by measuring cell viability with the Trypan Blue exclusion test (expressed as a percentage of viable cells with respect to the control cells). Another aliquot of cells was used to assess the genotoxicity of FW by the comet assay, according to the procedure previously described (26). Briefly, cell suspension was centrifuged (100 × g, for 3 min), re-suspended in 1% low-melting point agarose, and spread on microscope slides previously covered with a 1% normal-melting point agarose layer. Embedded cells were lysed, DNA was allowed to unwind, and then electrophoresis was performed. Then slides were washed with neutralization buffer, stained with ethidium bromide, and analyzed using a fluorescence microscope (BX60 Olympus, Japan) equipped with the Image-Pro Plus software (Immagini & Computer, Bareggio. Milano, Italy). Fifty images were analyzed for each slide, and the DNA damage was expressed as percentage of DNA in the tail.

### Statistical Analysis

Variables were expressed as median and interquartile range. Due to the paired data and small sample size, comparisons of variables before and after 1 month of KD were performed by the Wilcoxon matched-pairs test. All calculations were performed using SPSS version 17.0 for Windows (SPSS, Inc., Chicago, IL, USA). A *p* < 0.05 was considered statistically significant.

## Results

### Subjects

We enrolled seven epileptic patients, collecting a fecal sample before and after 1 month of KD. Adherence to the KD protocol was documented by constant ketonemia in all subjects. All participants completed the protocol, tolerated the diet, and there were no adverse effects. An improvement was reported by all patients, i.e., >50% reduction in seizures and involuntary movements.

### Ketogenic Dietary Treatment

Table 1 shows the daily dietary intake before and after the KD: the total energy intake and protein intake did not differ significantly while other macronutrients changed significantly. In particular, the fat content increased significantly from 54.6 (IQR 40.9–92.9) g/day to 125.7 (IQR 112.8–159.1) g/day (*p* = 0.028), the carbohydrate content diminished significantly from 130.0 (IQR 91.6–176.2) g/day to 20.2 (IQR 12.0–31.8) g/day (*p* = 0.018) and a significant reduction in dietary fiber content (*p* = 0.028) was found.

**Table 1:** Daily dietary intake before and after the treatment with KD.

	**Pre intervention (T0)**	**Post intervention (T1)**	
	**Median (IQR)**	**Median (IQR)**	***p***
Energy intake (kcal/day)	1278.0 (1115.0–1765.0)	1615.0 (1200.0–1675.0)	0.612
Energy intake (kcal/kg)	65.6 (20.6–77.0)	65.6 (28.3–75.4)	0.735
Protein (g/day)	56.2 (35.9–63.3)	36.0 (17.2–58.4)	0.310
Protein (% energy)	15.9 (10.2–19.4)	8.6 (5.8–13.9)	0.176
Fat (g/day)	54.6 (40.9–92.9)	125.7 (112.8–159.1)	*0.028*
Fat (% energy)	38.6 (34.3–49.0)	86.6 (78.1–88.3)	*0.018*
Saturated fat (% energy)	10.5 (9.2–15.3)	19.3 (14.6–21.9)	*0.042*
Monounsaturated fat (% energy)	10.1 (8.8–15.6)	19.3 (17.5–31.0)	*0.028*
Polyunsaturated fat (% energy)	3.2 (2.3–5.9)	10.3 (5.9–13.4)	*0.018*
Carbohydrates (g/day)	130.0 (91.6–176.2)	20.2 (12.0–31.8)	*0.018*
Carbohydrates (% energy)	40.3 (28.1–51.7)	4.3 (4.0–9.7)	*0.018*
Fiber (g/day)	12.8 (8.3–14.9)	7.4 (6.9–8.1)	*0.028*

*Data are expressed as median (interquartile range)*.

### SCFA Production

Concentrations of SCFA, expressed as mg/g dry weight feces, before and after KD, are reported in Table 2. We found a significant statistical difference before (T0) and after (T1) the KD for total SCFA, acetate, butyrate, propionate, and isobutyrate (*p* < 0.05), while iso-valerate was not significantly different between T0 and T1. Overall, the results showed that the KD strongly decreased the amount of SCFA in these patients.

**Table 2:** Fecal SCFA concentrations (mg/g dry feces) before (T0) and after 1 month (T1) of the KD.

	**T0**	**T1**	
	**Median (IQR)**	**Median (IQR)**	***p***
Total SCFA	20.7 (11.0–25.8)	9.3 (5.5–13.9)	*0.018*
Acetate	8.4 (4–12.2)	2.7 (2.1–5.3)	*0.018*
Butyrate	4.8 (3.8–6.8)	3.2 (1.3–4.2)	*0.018*
Propionate	3.4 (2.6–5.2)	2.4 (1.0–3.5)	*0.043*
Iso-valerate	1.1 (0.4–1.5)	0.6 (0.5–0.7)	0.128
Iso-butyrate	0.6 (0.4–1.0)	0.3 (0.2–0.4)	*0.028*

*Data are expressed as median (interquartile range)*.

### Fecal Water Toxicity

The toxicity of fecal water, a parameter of gut human health, was analyzed in our group of seven patients. First, we measured the cytotoxicity by using the Trypan Blue assay (Table 3): only one fecal water sample was cytotoxic, as the percentages of cell viability were around 50% (T0: 50.7 and T1: 43.1%). Additionally, we did not find any statistically significant difference of cytotoxicity before and after the KD for each subject. Afterward, we measured the fecal water genotoxicity with the comet assay (Table 3). As expected, the subject with high cytotoxicity showed the highest genotoxicity, both before and after the KD (T0: 64.9% and T1: 61.4% of DNA in the tail). The other subjects showed a medium level of genotoxicity, following the classification of Venturi et al. (17) (ranging between 25.3 and 41.8%). The median level of genotoxicity significantly decreased after the ketogenic therapy, therefore KD could have an impact on the human intestinal environment.

**Table 3:** Cytotoxicity (expressed as a percentage of viable cells) and genotoxicity (expressed as % of DNA in the tail) of fecal water before (T0) and after 1 month (T1) of the KD.

	**T0**	**T1**	
	**Median (IQR)**	**Median (IQR)**	***p***
Cytotoxicity	70.7 (61.0–84.8)	73.4 (71.3–79.8)	0.735
Genotoxicity	33.4 (32.0–41.8)	29.2 (26.2–32.6)	*0.018*

*Data are expressed as median (interquartile range)*.

## Discussion

A rich microbial ecology promotes the fundamental cross-talk and cross-feeding between species that guarantees the resilience of the gut ecosystem; conversely, one of the features of intestinal dysbiosis is the loss of diversity (27). Our study evaluated the effect of KD on SCFA and fecal water toxicity in seven patients, by collecting fecal samples before and after 1 month of dietary treatment.

A growing number of reports suggest that KD might alter human gut microbiota. Despite the different methods used (selective PCR or 16s rRNA gene sequencing), the low number of subjects involved, different age and country of residence, and different dietary treatment timing, the results of human studies (6–10) displayed that KD reduced the relative abundance of some taxa (i.e., Actinobacteria) and increased others (i.e., Bacteroidates). A similar outcome was observed in a murine model of autism spectrum disorder (3), which showed an anti-microbial-like effect of the KD by significantly decreasing the total host bacterial abundance.

The changes of microbiota can alter the microbiome, and modify the quality and levels of microbial metabolites, such as SCFA. To our knowledge, this is the first study that measured the SCFA concentrations after ketogenic diet treatment. The results showed that 1 month of dietary intervention significantly decreased total SCFA concentrations, particularly acetate, propionate, and butyrate; branched chain fatty acids were also reduced, but only isobutyrate was statistically significant. Comparing the basal SCFA levels with previous data obtained in healthy subjects, no significant differences were evident excluding a trend toward decreased acetate level (24). These important results indicate that the change in bacterial metabolite production may be caused by a specific impact of the ketogenic protocol, besides the pathological condition. It is worth noting that SCFA exert multiple beneficial effects on human metabolism (glucose and cholesterol metabolism, as well contributing to gut health) (16). Thus, impaired SCFA production might be detrimental for human health either at the local level, i.e., by altering the gut barrier permeability, or the systemic level, i.e., by altering the host immune system. Actually, the reduction of SCFA has been shown in studies investigating gut microbiota in different neurological pathologies (28) and systemic inflammatory response syndrome (29). The decrease in SCFA concentrations observed can be explained as a result of altered substrates availability during KD or as a consequence of the reduction of some microbial species to be able to ferment non-digestible carbohydrates. It is recognized that non-digestible carbohydrate availability in the large intestine can shape the whole microbial community by impacting on both the activity and the abundance of different bacterial groups able to produce SCFA (30–32).

Finally, as diet can affect the composition of human feces, modifying compounds in the water fraction and thus toxicity of fecal water—a risk factor for mucosa (33, 34)—we evaluated the cytotoxicity/genotoxicity of fecal water to better understand the global impact of the KD on the human gut.

The risk of colon cancer is increased by diet high in fat (35) mainly due to higher secretion of bile salts into the small intestine for fat digestion and absorption. The bile salts that escape ileum recycling, reach the colon where microbiota convert them into secondary bile acids. One of the most predominant secondary bile acids is deoxycholic acid, whose cancer-promoting activity was demonstrated in an animal model (36). Actually, higher secondary bile acids concentrations were found in colon fluids of humans with colon cancer (37), and healthy subjects tripled their bile acids concentrations in fecal water after adding a high amount of fat to their diet (38). A study *in vitro* on human colon adenocarcinoma cells confirmed the genotoxicity of secondary bile acids after exposure to physiological concentrations (20, 39). As KD intake of fat is consistent with the high fat diet protocols of Stadler's study (38), we should have expected detrimental effects on fecal water toxicity isolated after KD treatment.

Owing to the fact that high inter-individual variability of fecal water activity in the population is well-known (40) and previous studies have suggested that the evaluation of dietary intervention on FW toxicity should be assessed within the same subject (20, 41), we performed the Trypan blue test and comet assay measurements using basal data as the reference value. The results showed that the majority of samples presented medium fecal water genotoxicity, consistently with data detected in healthy Italian people (19). Only one subject displayed the highest cytotoxicity and consequently genotoxicity. This result, although suggesting a possible altered condition of intestinal environment, is in accordance with the published data, reporting that 15% of Italians are classified with high FW toxicity (19). Moreover, we found a statistically significant decrease in genotoxicity level after 1 month of KD. Although surprisingly, the results should be attributed to the strict control of dietary intake, consequently to the adoption of the KD, that favors gut well-being; otherwise the overall better health condition of these patients after KD, should have positively influenced the gut environment. In this study, we found that the classic KD seemed able to affect the gut environment by altering metabolites production. Indeed, we found lower SCFA levels after 1 month of KD, probably due to the low fermentable carbohydrate intake with KD or the reduction of fermenting bacteria, without adverse effects on FW cyto- and geno-toxicities. The obtained results sustain the hypothesis of the multistep impact of the KD on human health, extending its influence, beside the gut environment, to a systemic level and justifying further studies.

Health care professionals should be educated on the important role of dietary choices in modulating gut microbiota for therapeutic purposes. The maintenance of a healthy gut microbiota, via nutrition and use of food supplements, could lead to the reduction of the metabolic risk associated with unbalanced diets (42). There are some limitations that need to be clarified. First, the small size and the wide age range of the group of the participants was due to the difficulties encountered during subject recruitment. Besides the rarity of the pathologies considered, most of the patients affected by drug-resistant epilepsy did not have fecal continence, thus they were not eligible for the study. Secondly, it would have been interesting to evaluate the effect of KD on the human gut after a further follow-up, as other studies (3, 7) have reported a re-establishment of the dysbiosis condition after several weeks of ketogenic dietary treatment. Despite these limitations, which must be considered when interpreting the results, this study suggests that KD has an impact on the human gut, highlighting the need for further research to better investigate the state of dysbiosis and optimize the therapy. For instance, it may be reasonable to suggest a supplementation of probiotics and/or prebiotics to potentially prevent microbiota dysfunction.

## Data Availability Statement

The original contributions presented in the study are included in the article, further inquiries can be directed to the corresponding authors.

## Ethics Statement

The studies involving human participants were reviewed and approved by Ethical Commitee of San Paolo Hospital—University of Milan Protocol number 2017/EM/147. Written informed consent to participate in this study was provided by the participants' legal guardian/next of kin.

## Author Contributions

DE and MC: conceptualization and project administration. MC and AT: supervision. VD, MC, and DE: resources. DE, EM, and CF: writing. EM and CF: investigation, data curation, and visualization. All authors contributed to the article and approved the submitted version.

## Conflict of Interest

The authors declare that the research was conducted in the absence of any commercial or financial relationships that could be construed as a potential conflict of interest.

## Abbreviations

KD: ketogenic diet
DRE: drug-resistant epilepsy
SCFA: short chain fatty acids
FW: fecal water.
